# Development and experimental validation of an energy metabolism-related gene signature for diagnosing of osteoporosis

**DOI:** 10.1038/s41598-024-59062-y

**Published:** 2024-04-08

**Authors:** Yao Lu, Wen Wen, Qiang Huang, Ning Duan, Ming Li, Kun Zhang, Zhong Li, Liang Sun, Qian Wang

**Affiliations:** 1 Department of Orthopaedics, Honghui Hospital, Xi’an Jiaotong University, 555 Youyi East Road, Xi’an, 710054 Shaan’xi Province China; 2 Department of Orthopedics, Hospital of Chengdu University of Traditional Chinese Medicine, Chengdu, China

**Keywords:** Osteoporosis, Energy metabolism, Risk model, Immune microenvironment, Computational biology and bioinformatics, Immunology, Biomarkers, Medical research

## Abstract

Osteoporosis is usually caused by excessive bone resorption and energy metabolism plays a critical role in the development of osteoporosis. However, little is known about the role of energy metabolism-related genes in osteoporosis. This study aimed to explore the important energy metabolism-related genes involved in the development of osteoporosis and develop a diagnosis signature for osteoporosis. The GSE56814, GSE62402, and GSE7158 datasets were downloaded from the NCBI Gene Expression Omnibus. The intersection of differentially expressed genes between high and low levels of body mineral density (BMD) and genes related to energy metabolism were screened as differentially expressed energy metabolism genes (DE-EMGs). Subsequently, a DE-EMG-based diagnostic model was constructed and differential expression of genes in the model was validated by RT-qPCR. Furthermore, a receiver operating characteristic curve and nomogram model were constructed to evaluate the predictive ability of the diagnostic model. Finally, the immune cell types in the merged samples and networks associated with the selected optimal DE-EMGs were constructed. A total of 72 overlapped genes were selected as DE-EMGs, and a five DE-EMG based diagnostic model consisting *B4GALT4, ADH4, ACAD11, B4GALT2*, and *PPP1R3C* was established. The areas under the curve of the five genes in the merged training dataset and B4GALT2 in the validation dataset were 0.784 and 0.790, respectively. Moreover, good prognostic prediction ability was observed using the nomogram model (C index = 0.9201; *P* = 5.507^e−14^). Significant differences were observed in five immune cell types between the high- and low-BMD groups. These included central memory, effector memory, and activated CD8 T cells, as well as regulatory T cells and activated B cells. A network related to DE-EMGs was constructed, including hsa-miR-23b-3p, DANCR, 17 small-molecule drugs, and two Kyoto Encyclopedia of Genes and Genomes pathways, including metabolic pathways and pyruvate metabolism. Our findings highlighted the important roles of DE-EMGs in the development of osteoporosis. Furthermore, the DANCR/hsa-miR-23b-3p/B4GALT4 axis might provide novel molecular insights into the process of osteoporosis development.

## Introduction

Osteoporosis is an age-related chronic bone disease characterized by low bone mineral density (BMD). This disease occurs widely in all racial groups and causes more than 8.9 million fractures per year. Moreover, the fracture risk continues to increase, especially in Asia^1,2^. Among elderly and middle-aged Chinese residents, 33.49% develop osteoporosis^3^. The disease commonly manifests in individuals aged 50 years and often goes unnoticed until a fracture occurs. Excessive bone resorption, leading to an imbalance in bone remodeling, has been a common reason for disease development. In addition, late diagnosis greatly increases the risk of poor overall bone health and growth, including osteoporotic fractures in primary areas such as the hip, spine, and wrist^4^. Therefore, there is an urgent need to identify improved treatment strategies and biomarkers for an early diagnosis.

Bone resorption is mainly controlled by the number and activity of osteoclasts. Similarly, osteoblasts are bone-forming cells that are important for skeleton maintenance and growth. The dynamic processes of energy generation have attracted considerable attention in recent years^5^. Anabolic treatments have been accepted for osteoporosis, leading to the further exploration of substrate utilization by osteoblasts^6^. Several pathways related to energy metabolism have been identified in osteoporosis. Wnt signaling is a critical mechanism for increasing aerobic glycolysis and bone accrual. Wnt10b, Wnt7b, and Wnt3a could promote osteoblast differentiation and stimulate lactate production and glucose consumption^7,8^. Moreover, the functions of mTORC and mTOR in bone formation, which contribute to nutritional coordination, have also been demonstrated^9^. Organ transplant patients receiving mTOR inhibitors as immunosuppressive agents have a higher incidence of osteoporosis^10^. However, there remains a limited understanding of the role of energy metabolism-related genes in bone.

**Figure 1:**
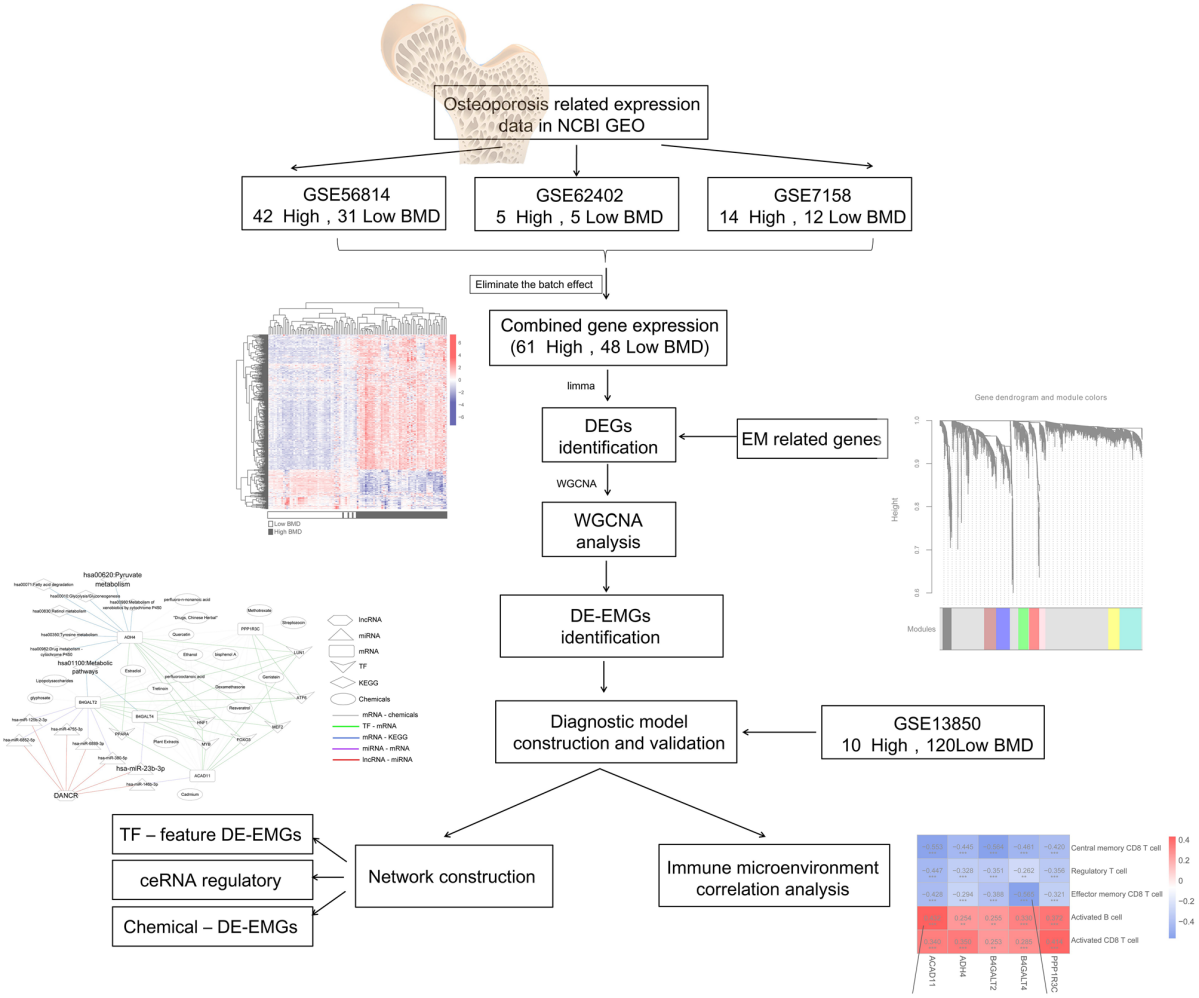
A flow chart of the present study.

This study combined expression profile data from multiple blood tissues with different levels of body mineral density (BMD). First, genes related to energy metabolism (EM) were obtained from the literature^11–13^, and EM genes related to osteoporosis were screened in the expression profile. Diagnostic EM genes and related immune microenvironment signatures were subsequently analyzed using optimization algorithms, and a disease diagnosis model was constructed. A flowchart of this study is shown in Fig. 1.

## Materials and methods

### Data source

Osteoporosis-related datasets, including GSE56814, GSE62402, and GSE7158^14–16^ were downloaded from the NCBI Gene Expression Omnibus (GEO; https://www.ncbi.nlm.nih.gov/)^17^. The GSE56814 dataset includes peripheral blood monocytes from 42 and 31 patients with high and low BMD, respectively. GSE62402 included peripheral blood monocytes from five patients with high BMD and five patients with low BMD. These two datasets were sequenced using the [HuEx-1_0-st] Affymetrix Human Exon 1.0 ST Array. GSE7158 included peripheral blood monocytes from 14 and 12 patients with high and low BMD, respectively. The data were based on the [HG-U133_Plus_2] Affymetrix Human Genome U133 Plus 2.0 Array.

### Selection of differentially expressed EM genes

The batch effects of the three datasets were first removed using the sva package (version 3.38.0, http://www.bioconductor.org/packages/release/bioc/html/sva.html)^18^ in R3.6.1. The combined expression levels were then calculated.

Differentially expressed genes (DEGs) of low and high BMD were screened using the limma package (version 3.34.7)^19^ in R3.6.1. The thresholds were set as FDR < 0.05 and |log_2_FC|> 0.263. Directional hierarchical clustering was performed using the pheatmap package (Version 1.0.8, https://cran.r-project.org/package=pheatmap) in R3.6.1 and visualized using a heatmap.

The intersection of DEGs and EM genes was selected as differentially expressed energy metabolism genes (DE-EMGs). The methylation levels of the DE-EMGs were also determined. Finally, gene ontology (GO) and Kyoto Encyclopedia of Genes and Genomes (KEGG) pathway enrichment analysis^20,21^ were performed using the DAVID software (version 6.8, https://david.ncifcrf.gov/)^22,23^. GO enrichment analysis was used to investigate the functions of the DE-EMGs, including biological processes (BP), cellular components (CC), and molecular functions (MF). *P* < 0.05 was defined as the threshold.

### Screening of disease-related genes based on WGCNA algorithm

Weighed gene co-expression network analysis (WGCNA) is a bioinformatic algorithm for constructing co-expression networks. The network could identify modules associated with diseases and screen important pathogenic mechanisms or potential therapeutic targets. Modules related to disease status were screened based on genes detected in the merged dataset using the WGCNA package (version 1.61, https://cran.r-project.org/web/packages/WGCNA/index.html)^24^ in R3.6.1. WGCNA was performed by defining adjacency functions and module partitioning. The threshold for module partitioning screening was set as follows: the module set contained at least 150 genes and cutHeight = 0.995.

The selected DE-EMGs were mapped onto each WGCNA module. A hypergeometric algorithm was used to calculate the fold enrichment parameter and enrichment significance *p*-value of the differential genes in the module using the following formula: f (k, N, M, n) = C (k, M) × C (n-k, N-M)/C (n, N)^25^. N represents all genes involved in the WGCNA of the algorithms, M represents the number of genes in each module obtained by the WGCNA algorithm, n represents the number of significantly differentially expressed genes filtered in Step 2, and k represents the number of significantly differentially expressed genes mapped to the corresponding module. Modules with *P* < 0.05 and Fold enrichment > 1 were selected. Finally, modules significantly enriched in DEGs and DE-EMGs involved in the modules were included for further analysis.

### DE-EMG-based diagnostic model

Single-factor logistic regression analysis was conducted using RMS (version 6.3-0, https://cran.r-project.org/web/packages/rms/index.html) in R3.6.1. DE-EMGs with *P* < 0.05 were included for further analyses. Furthermore, optimal DE-EMGs were screened using the LASSO algorithm in the Lars package (version 1.2, https://cran.r-project.org/web/packages/lars/index.html)^26^ from R3.6.1.

A diagnostic model based on the optimal DE-EMGs was constructed using a support vector machine in R3.6.1 e1071 (Version 1.6-8, https://cran.r-project.org/web/packages/e1071) on the merged training set. The receiver operating characteristic (ROC) curve of the merged training dataset and independent validation dataset (GSE13850) was constructed using R 3.6.1, pROC (Version 1.12.1, https://cran.r-project.org/web/packages/pROC/index.html)^27^ to verify the efficiency of the diagnostic model.

The alignment diagram, also known as the nomogram, integrates multiple predictive indicators using multivariate regression analysis. Here, these indicators were visualized on the same plane at a certain scale using scaled line segments to evaluate the interrelationships among the various variables in the predictive model^28^. The nomogram model and correct line chart were constructed using the RMS package (version 5.1-2; https://cran.r-project.org/web/packages/rms/index.html) in R3.6.1.

### Evaluation of immune features based on the ssGSEA algorithm

The microenvironment comprises fibroblasts, immune cells, the extracellular matrix, growth factors, inflammatory factors, and special physicochemical characteristics. Microenvironments significantly affect the diagnosis, survival outcomes, and clinical treatment sensitivity of diseases. Cells in the microenvironment can aggregate into different categories. In addition, there are complex and significant interactions between each cell type and other cells, as well as the infiltration patterns of some robust cells. Immunological signature gene sets were downloaded from the gene set enrichment analysis database (GSEA, http://software.broadinstitute.org/gsea/index.jsp) to evaluate the immune infiltration types in the merged samples. Next, the immune infiltration types of the merged samples were evaluated using the GSVA package (Version 1.36.3)^29^ in R3.6.1. This was determined based on single-sample GSEA (ssGSEA, http://www.bioconductor.org/packages/release/bioc/html/GSVA.html). The proportion of immune cells in the low- and high-BMD samples was compared using the Kruskal–Wallis test. Finally, the correlation between the expression levels of the optimized DE-EMGs used to construct the diagnostic models and important immune cells was calculated using the COR function in R3.6.1.

### Network construction based on DE-EMGs

#### Construction of TF regulatory relationships

Related TFs targeting DE-EMGs were explored using transcriptional regulatory relationships revealed by sentence-based text mining (TRRUST, https://www.grnpedia.org/trrust/)^30^. All the related TFs and their target genes were downloaded from the database. The DE-EMGs selected for diagnostic model construction and related TFs were screened for further studies.

#### Construction of the ceRNA network

The DE-EMGs selected for diagnostic model construction and related miRNAs were searched using miRWalk 3.0 (http://129.206.7.150/)^31^. Then, lncRNAs related with the selected miRNA were explored using DIANA-LncBasev2 (http://carolina.imis.athena-innovation.gr/diana_tools/web/index.php?r=lncbasev2%2Findex-experimental)^32^. Similarly, lncRNAs related to osteoporosis were explored using lncRNA Disease (http://www.cuilab.cn/lncrnadisease)^33^. Next, an lncRNA-miRNA-mRNA network related to osteoporosis was constructed.

#### Construction of chemical molecules and KEGG related to osteoporosis

Chemical drug molecules and KEGG pathways were explored using the Comparative Toxicogenomics Database 2023 update (http://ctd.mdibl.org/)^34^. Thereafter, chemical drug molecules targeted by the DE-EMGs were selected for the diagnostic model. Finally, an integrated network of DE-EMGs was constructed for diagnostic models.

### Reverse-transcription quantitative PCR (RT-qPCR)

Fresh peripheral blood was collected from eight low-BMD people (6 females, 2 males) and eight high-BMD people (4 females, 4 males). Total RNA from monocytes was extracted by Trizol reagent (GENSTAR Inc. Beijing, China) and was reverse transcribed to cDNA with Primer ScriptTM RT Reagent (Thermo Fisher Scientific, Waltham, MA, USA). Then, real-time PCR was performed on a RT-qPCR machine (Bio-Rad, Hercules, CA, United States) with a SYBR green detection system (TargetMol Chemicals Inc. Shanghai, China). GAPDH was used as internal control. The primers used are listed in Table 1. Written and informed consent was obtained from all participants. The study was approved by the Ethics Committee of Honghui Hospital, Xi’an Jiaotong Universty.

**Table 1:** The primers used for RT-qPCR.

Gene	Direction	Sequence (5′–3′)
B4GALT4	Forward	CTCTGACTAATGAAGCATCCACG
B4GALT4	Reverse	CTGCCTGTACCTCTTCCAAAGTG
ADH4	Forward	CCTTGACTGTGCAGGTGGATCT
ADH4	Reverse	GTCAATCCTTTGCTACCAGCAGC
ACAD11	Forward	GTGCAACCTCTGGCAGAAACTG
ACAD11	Reverse	CCTGACCTTTCCGAGTCTGTAC
B4GALT2	Forward	GACCGCGACAAGCATAACGAAC
B4GALT2	Reverse	AGACACCTCCAAGACCTGGTAC
PPP1R3C	Forward	CCTCTGCCTTAAAACACCACGAG
PPP1R3C	Reverse	CAACGAGCAGTTCTCCAGACAG
GAPDH	Forward	GTCTCCTCTGACTTCAACAGCG
GAPDH	Reverse	ACCACCCTGTTGCTGTAGCCAA

### Statistical analysis

The bioinformatics analysis was performed by corresponding packages in R3.6.1. *P* < 0.05 or FDR < 0.05 was regarded as threshold for statistical significance level when applicable. For the experimental validation, data were analyzed by student’s *t* test in GraphPad Prism 9.0 (Boston, MA, USA) with significance level of *P* < 0.05.

### Ethics approval and consent to participate

Written and informed consent was obtained from all participants. The study was approved by the Ethics Committee of Honghui Hospital, Xi’an Jiaotong Universty.

## Results

### Selection of DE-EMGs

**Figure 2:**
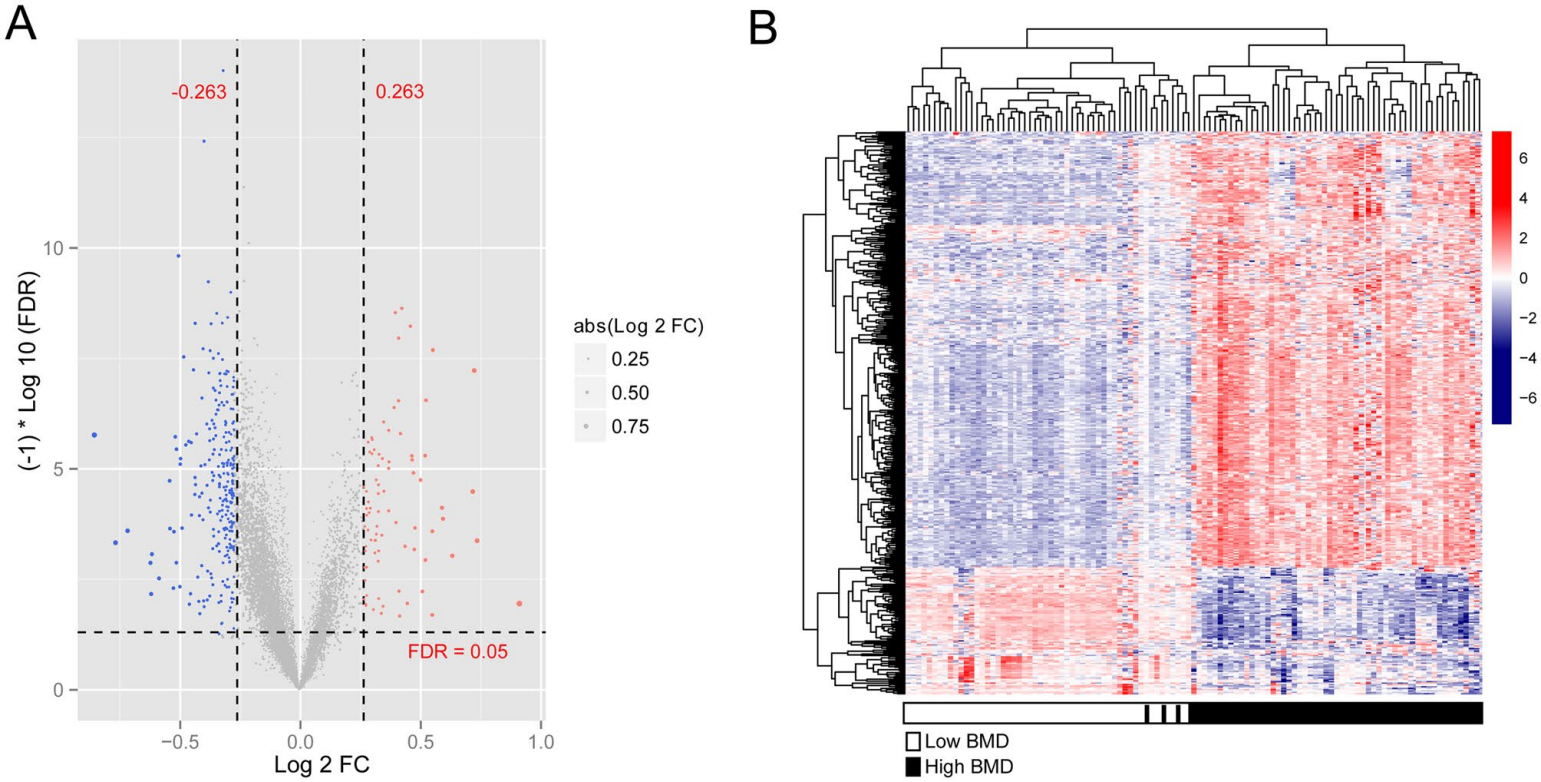
Differentially expressed genes (DEGs) between high- and low-body mineral density (BMD). (**A**) Volcano plot of DEG selection. The blue and red dots represent significantly downregulated and upregulated DEGs, respectively. The black horizontal line represents a fold discovery rate (FDR) < 0.05, and the two vertical lines represent a | log2 fold change (FC) |> 0.263; (**B**) The heatmap of DEGs. Black and white bars represent the high- and low-BMD groups, respectively.

Batch effects of the three datasets were removed. Next, the datasets were merged into one. The participants were then divided into high- and low-BMD groups, which included 61 and 48 samples, respectively. A total of 427 DEGs with high vs. low BMD were obtained. A volcano plot of DEGs is shown in Fig. 2A. A heatmap of DEGs showed different expression levels of DEGs in participants with high BMD compared with those with low BMD (Fig. 2B).

**Figure 3:**
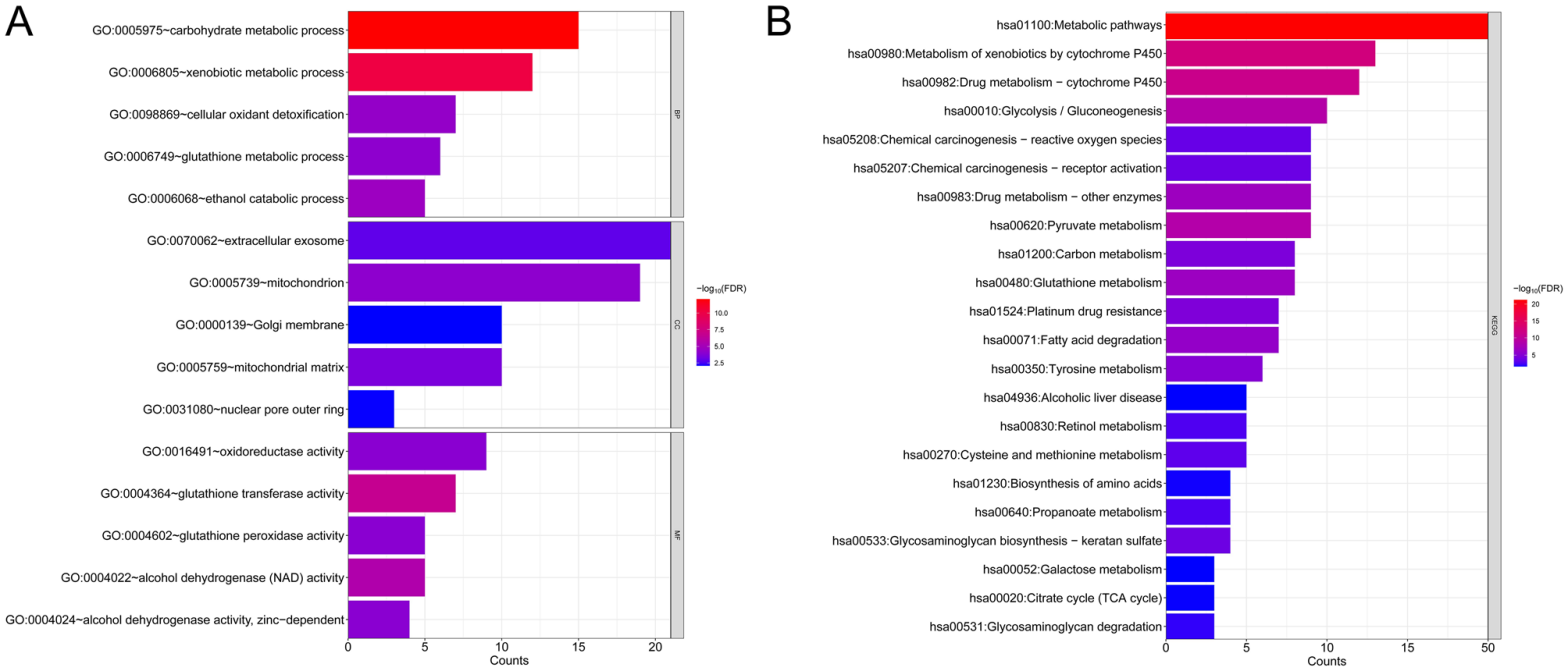
Functional enrichment analysis of differentially expressed energy metabolism genes (DE-EMGs). (**A**) The top 15 gene ontology items enriched by DE-EMGs; (**B**) Kyoto Encyclopedia of Genes and Genomes (KEGG) pathways enriched by DE-EMGs.

A comparison of the EM-related genes with DEGs revealed 72 overlapping genes that were identified as DE-EMGs. Furthermore, GO and KEGG pathway enrichment analysis were performed. Briefly, the identified DE-EMGs were significantly enriched in 43 GO terms (21 BPs, 10 CCs, 12 MFs), such as carbohydrate metabolic, xenobiotic metabolic, and ethanol catabolic processes (Fig. 3A). In contrast, 22 KEGG pathways, including metabolic pathways, xenobiotic metabolism by cytochrome P450, and drug metabolism by cytochrome P450, were enriched in these DE-EMGs (Fig. 3B).

### Screening of osteoporosis-related genes based on the WGCNA algorithm

The value of the adjacency matrix weight parameter power was explored to satisfy the precondition of a scale-free network distribution. The range of the network construction parameters was first selected, and then the scale-free distribution topology matrix was calculated.

**Figure 4:**
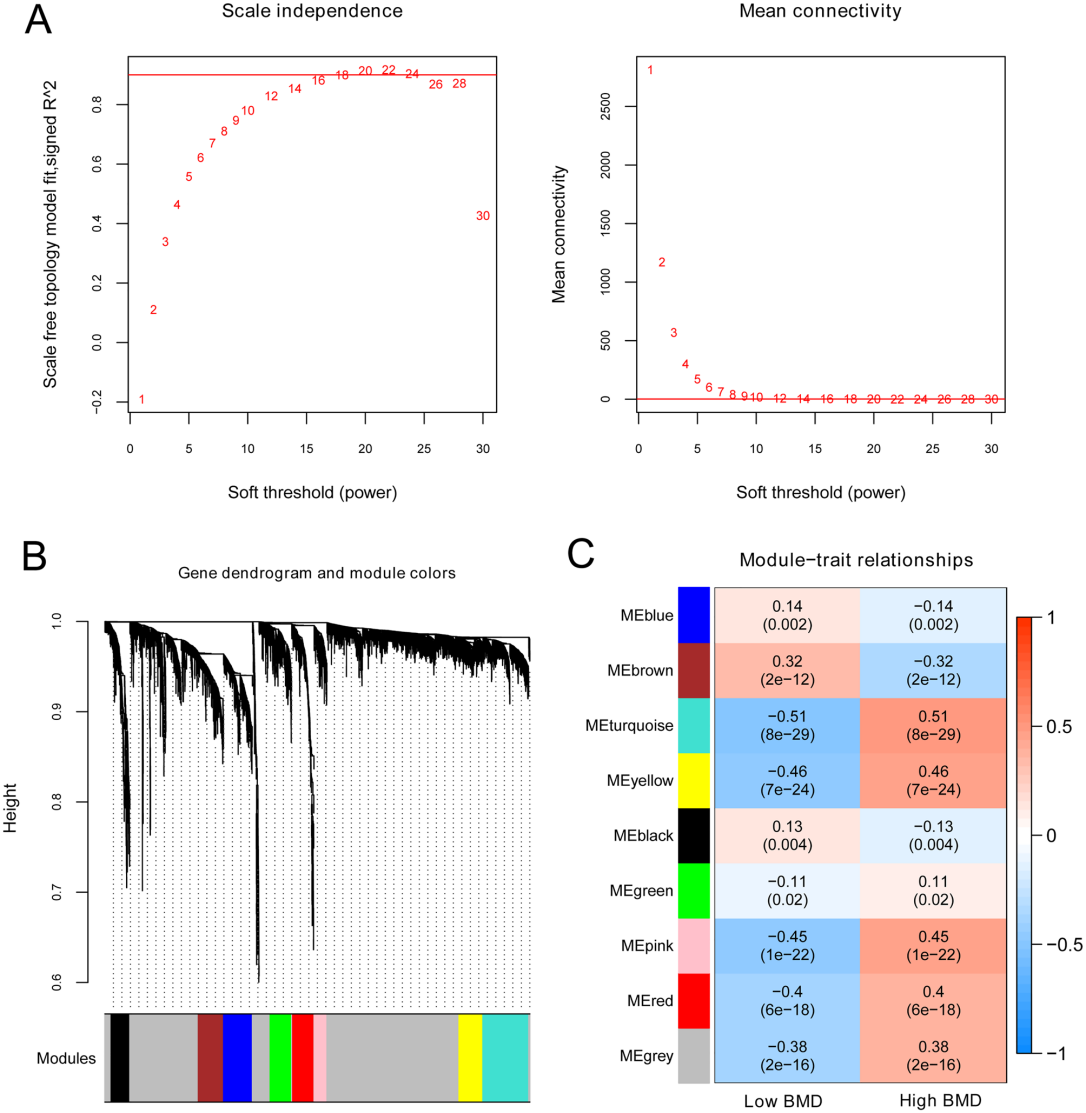
Screening of osteoporosis-related genes based on the WGCNA algorithm. (**A**) An adjacency matrix weight parameter power selection graph and the schematic diagram of average connectivity of genes under different power parameters. The horizontal axis represents the weight parameter power, whereas the vertical axis represents the square of the correlation coefficients between log (k) and log (*p* (k)) in the corresponding network. The red line represents the standard line where the square value of the correlation coefficient reaches 0.9. The red line in the right graph indicates the average connectivity of network nodes under the weight parameter power of the adjacency matrix in the left figure; (**B**) A module division tree diagram, with each color representing different modules; (**C**) A module-trait related heatmap.

As shown in Fig. 4A, the value of power was selected when the square value of the correlation coefficient reached 0.9 for the first time (power = 18). The average node connectivity of the constructed co-expression network was 1, which fully conformed to the properties of small-world networks. The dissimilarity coefficient between gene points was then calculated, and a system clustering tree was obtained. The minimum number of genes for each module was set to 150, and the cut height was 0.995. Nine modules were obtained (Fig. 4B). The correlation between the BMD status of the samples and each module is shown in Fig. 4C. MEblue, MEbrown, and MEblack positively correlated with low BMD and negatively correlated with high BMD. The other five modules, namely MEturquoise, MEyellow, MEpink, MEred, and MEgrey, were positively correlated with high BMD and negatively correlated with low BMD.

Here, 354 DEGs were mapped to the modules. As shown in Table 2, 60 DEGs were significantly enriched in the MEturquoise module and 12 DE-EMGs were included namely: ABCC5, ACAD11, ADH4, ADH6, B3GAT3, B4GALT2, B4GALT4, MECR, PC, PGK2, PHKG1, and PPP1R3C.

**Table 2:** The information on modules.

ID	Color	Module size	#DEGs	Enrichment information	
				Enrichment fold [95%CI]	P_hyper_
Module 1	Black	254	5	0.321 [0.103–0.765]	5.39E−03
Module 2	Blue	393	10	0.414 [0.195–0.779]	3.35E−03
Module 3	Brown	342	16	0.762 [0.426–1.273]	3.49E−01
Module 4	Green	292	1	0.0557 [0.00141–0.315]	1.28E−06
Module 5	Grey	3075	205	1.085 [0.904–1.300]	3.85E−01
Module 6	Pink	173	12	1.129 [0.567–2.049]	6.33E−01
Module 7	Red	289	18	1.014 [0.585–1.655]	9.01E−01
Module 8	Turquoise	621	60	1.573 [1.161–2.101]	2.98E−03
Module 9	Yellow	324	27	1.357 [0.867–2.045]	1.60E−01

**Figure 5:**
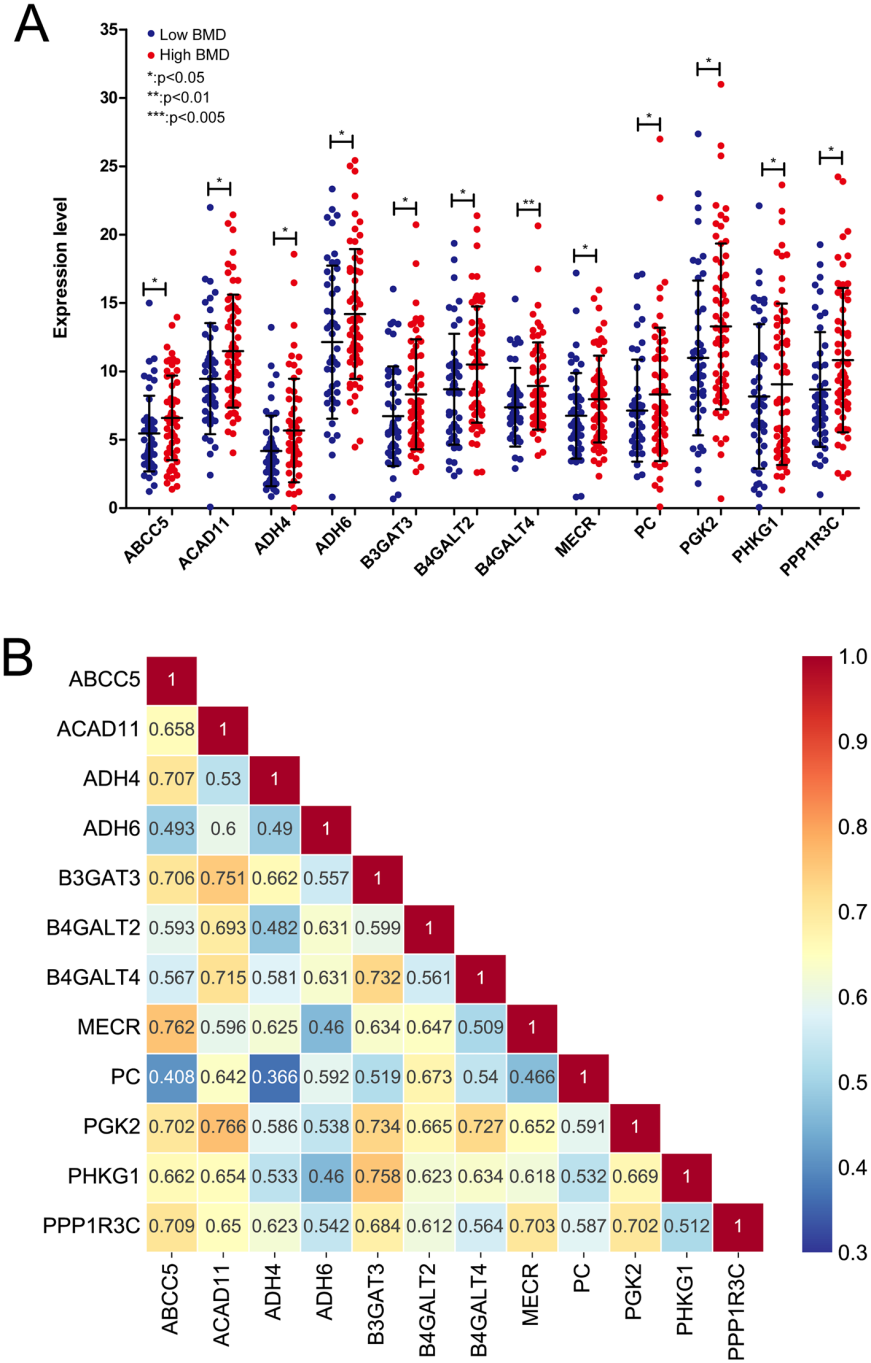
The expression levels of 12 DE-EMGs between high- and low-BMD. (**A**) Comparison of 12 DE-EMG expression levels between high and low BMD; (**B**) A heatmap display of the correlation of expression levels of 12 DE-EMGs.

The expression levels of the 12 DE-EMGs in high- and low-BMD groups are shown in Fig. 5A. Here, the expression levels of DE-EMGs in the high-BMD group were all significantly higher than those in the low-BMD group (*P* < 0.05). The relationships among these 12 DE-EMGs are shown in Fig. 5B.

### Construction and evaluation of the diagnostic model

**Figure 6:**
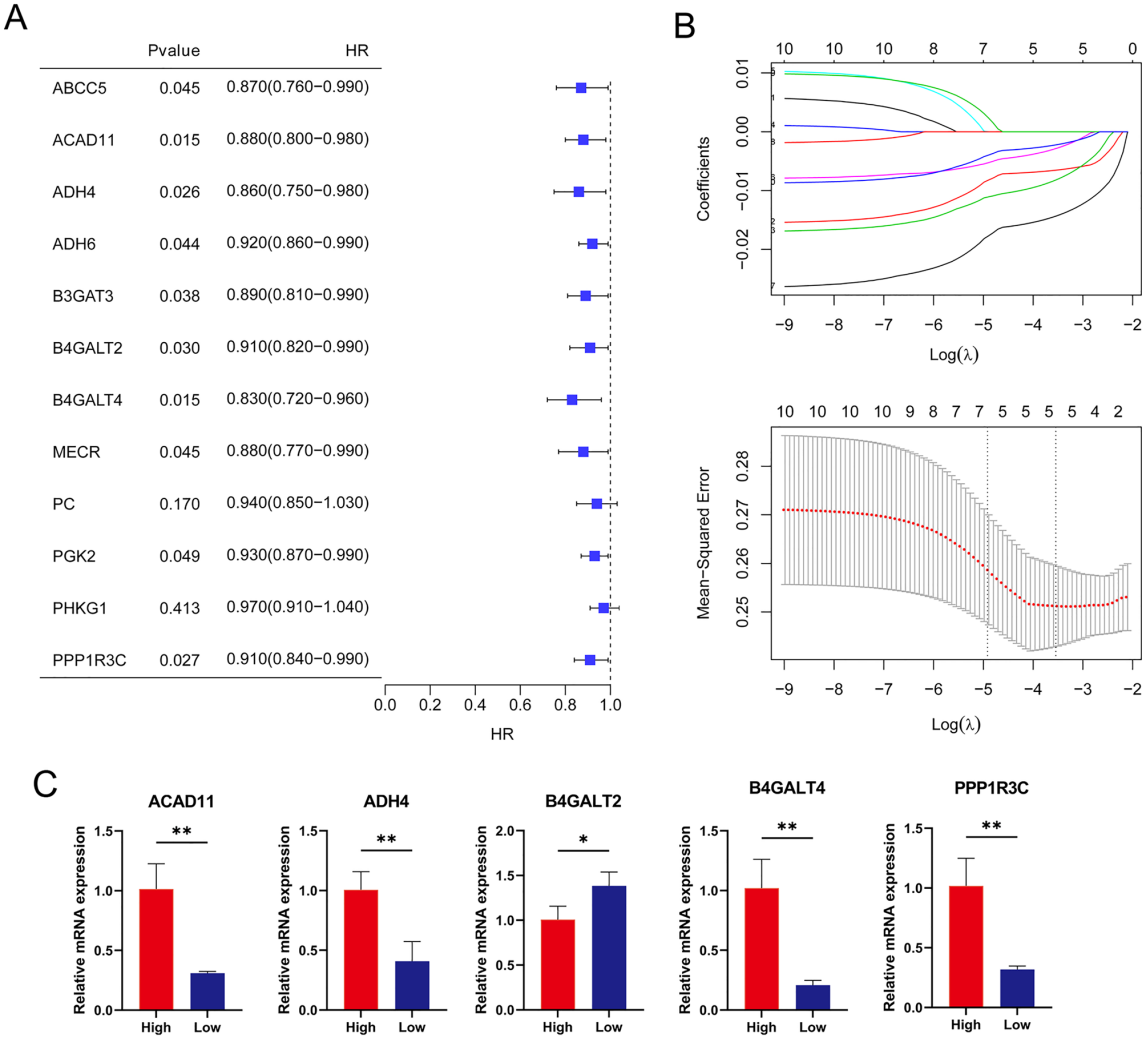
Single-factor logistic regression analysis based on expression levels of 12 DE-EMGs. (**A**) The results of single-factor logistic regression analysis; (**B**) A LASSO parameter graph. (**C**) Experimental validation of five DE-EMGs in the diagnosis model. *, ** indicates *P* < 0.05 and *P* < 0.01.

Single-factor logistic regression analysis was performed to explore genes related to the survival performance of osteoporosis. Ten DE-EMGs were significantly related to the survival performance of osteoporosis (Fig. 6A; *P* < 0.05). The optimal DE-EMG combination was further analyzed using least absolute shrinkage and selection operator (LASSO) in the lars package in R3.6.1. The parameters of LASSO are shown in Fig. 6B. Finally, five DE-EMGs were selected as optimal for diagnosis, namely: B4GALT4, ADH4, ACAD11, B4GALT2, and PPP1R3C. The differential expression of the five DE-EMGs were further validated by qRT-PCR in our cohort of low-BMD and high-BMD patients. As shown in Fig. 6C, the expression levels of B4GALT4, ADH4, ACAD11 and PPP1R3C were significantly increased (*P* < 0.01), while B4GALT2 was significantly decreased in high-BMD group (*P* < 0.05). The experimental validation results showed a relatively high consistency rate with bioinformatics study.

**Figure 7:**
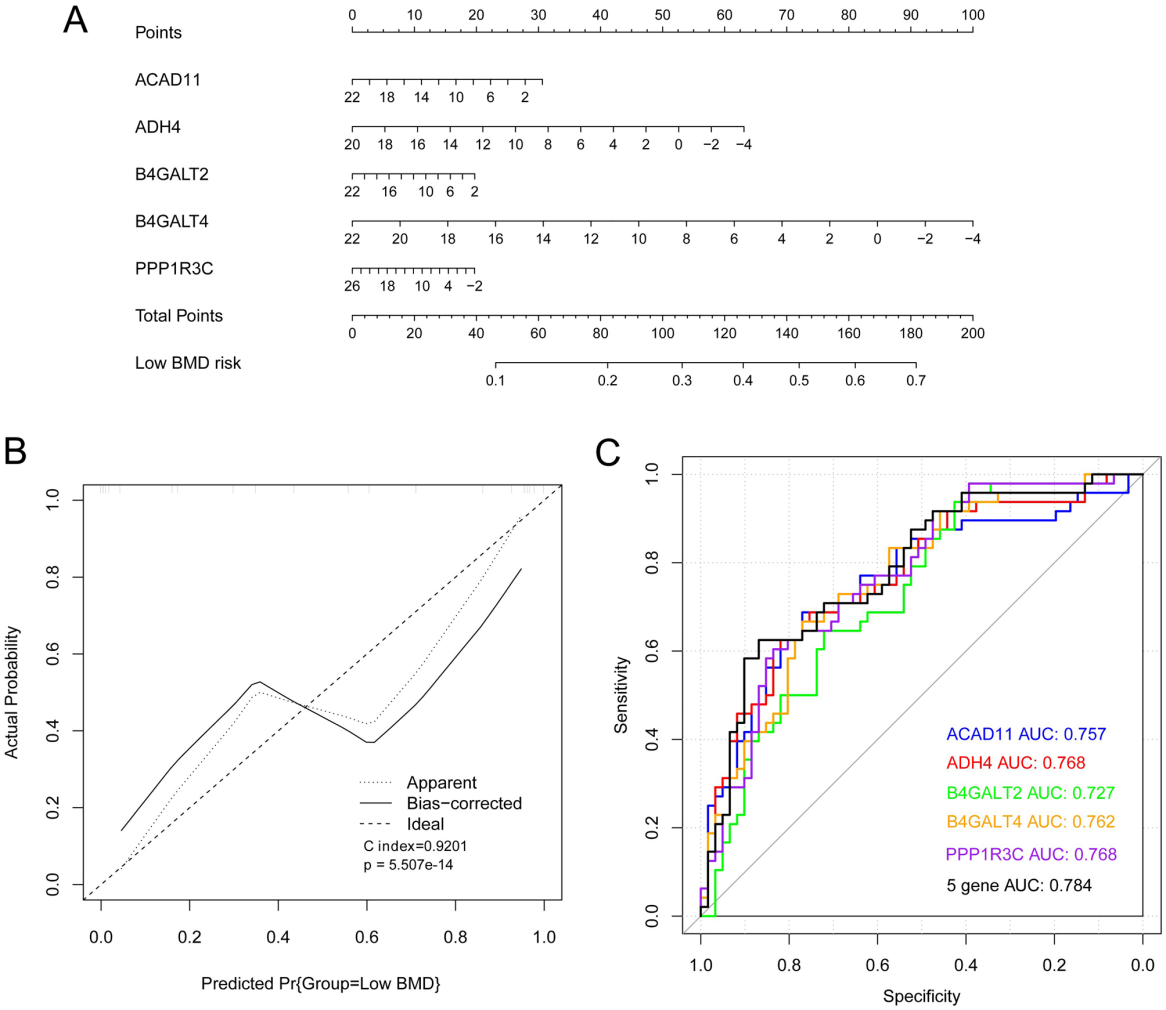
A nomogram model and corrected curves based on data from the merged training dataset. (**A**) Nomogram of five DE-EMGs selected for constructing the diagnosis model; (**B**) A corrected line chart; (**C**) A receiver operating characteristic (ROC) curve.

A nomogram model (Fig. 7A) and corrected curves (Fig. 7B) were constructed to evaluate the efficiency of the diagnostic model based on the data from the merged training dataset. B4GALT4 made the greatest contribution to survival. Good predictive ability was observed in the model (C-index = 0.9201; *P* = 5.507^e−14^). Furthermore, the areas under the curve (AUC) of the five genes was 0.784, which had the highest predictive value, followed by ADH4 (AUC = 0.768), PPP1R3C (AUC = 0.768), B4GALT4 (AUC = 0.762), ACAD11 (AUC = 0.757), and B4GALT2 (AUC = 0.727; Fig. 7C).

**Figure 8:**
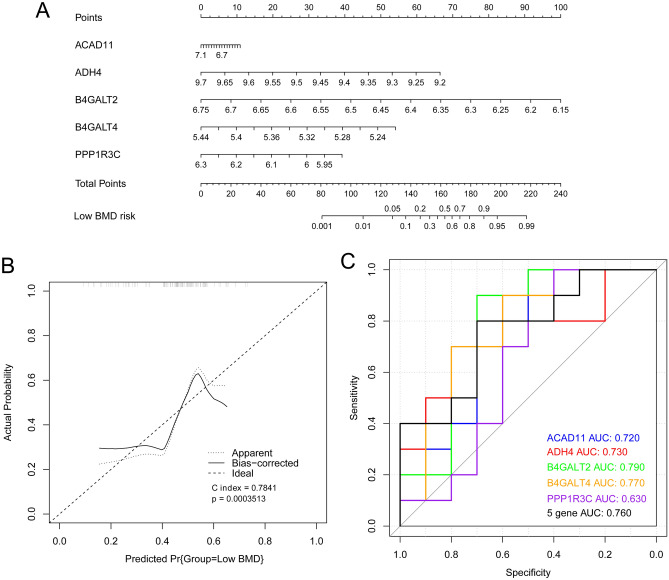
A nomogram model and corrected curves based on data from the GSE13850 validation dataset. (**A**) A nomogram of five DE-EMGs selected for constructing the diagnosis model; (**B**) The corrected line chart; (**C**) An ROC curve.

A nomogram model (Fig. 8A) and corrected curves (Fig. 8B) were constructed to evaluate the efficiency of the diagnostic model based on the validation dataset. B4GALT2 made the greatest contribution to survival. The predictive ability of the model showed similar performance to the ideal model (C-index = 0.7841, *P* = 0.0003513). ROC analysis revealed that the AUC of B4GALT2 was 0.790. This had the highest predictive value, followed by B4GALT4 (AUC = 0.770), 5 gene (AUC = 0.760), ADH4 (AUC = 0.730), ACAD11 (AUC = 0.720), and PPP1R3C (AUC = 0.630; Fig. 8C). The specificity, sensitivity and Youden index is displayed in Table 3.

**Table 3:** The specificity, sensitivity and Youden index in training set and validation set.

ID	Training set			Validation set		
	Specificity	Sensitivity	Youden index	Specificity	Sensitivity	Youden index
ACAD11	0.691	0.733	0.424	0.662	0.711	0.373
ADH4	0.702	0.743	0.445	0.672	0.721	0.392
B4GALT2	0.664	0.704	0.368	0.727	0.78	0.507
B4GALT4	0.696	0.738	0.434	0.708	0.76	0.469
PPP1R3C	0.702	0.743	0.445	0.611	0.622	0.233
5 gene	0.716	0.759	0.475	0.699	0.751	0.45

### Immune characteristics based on ssGSEA

**Figure 9:**
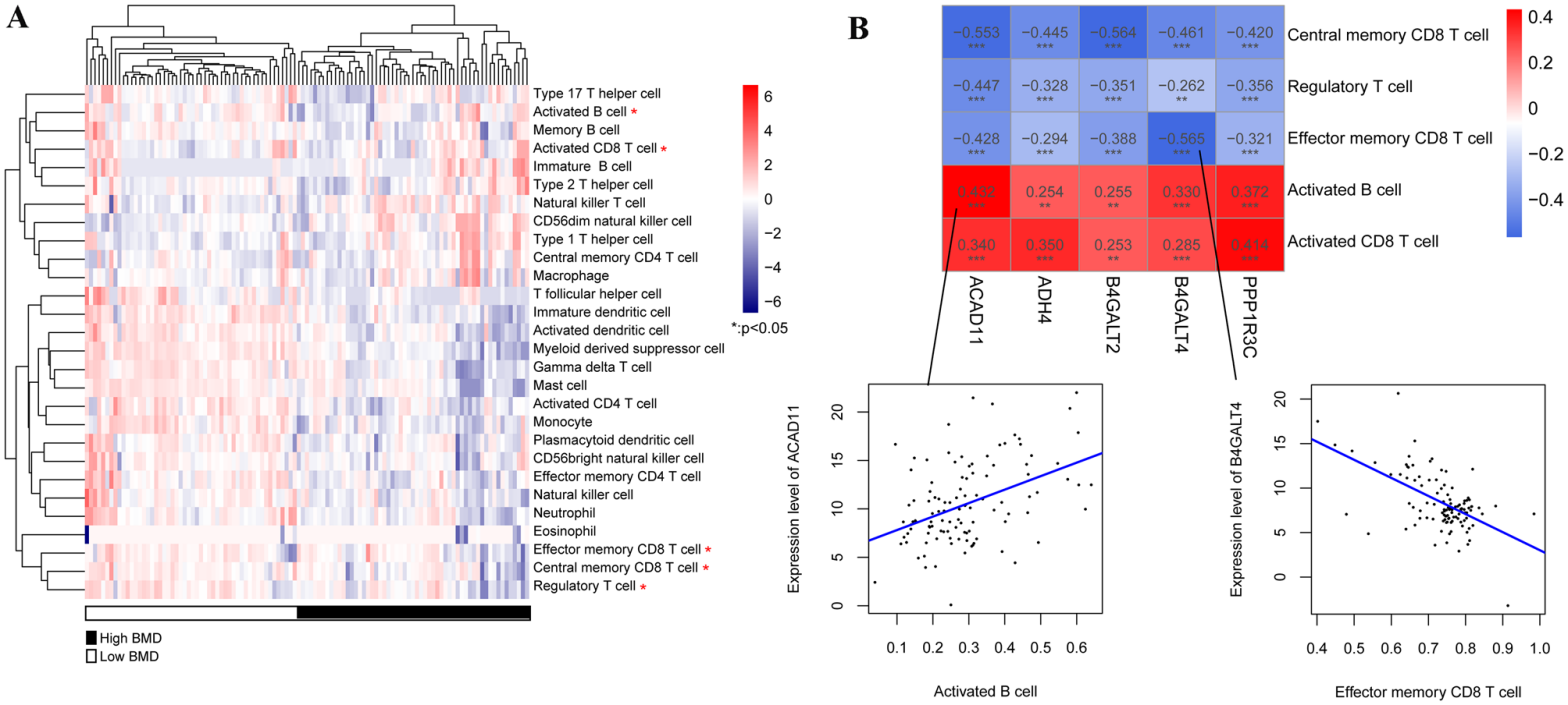
Evaluation of sample immune features based on the ssGSEA algorithm. (**A**) A heatmap of 28 immune cell type distribution in high- and low-BMD groups; (**B**) The correction between five DE-EMGs selected for constructing a diagnosis model and immune cells.

The ratios of 28 immune cells were determined using ssGSEA. A heat map of the 28 immune cell distributions between the high- and low-BMD groups is shown in Fig. 9A. Significant differences in five immune cell types were observed between the high- and low-BMD groups, including central memory, effector memory, and activated CD8 T cells, as well as regulatory T cells and activated B cells. The relationship between the five selected DE-EMGs and immune cells was further analyzed (Fig. 9B). Activated B cells and CD8 T cells were positively correlated with the five selected DE-EMGs. Central memory CD8 T cells, regulatory T cells, and effector memory CD8 + T cells were negatively correlated with the five selected DE-EMGs.

### Network correlated with DE-EMGs

Seven TFs related to the five selected DE-EMGs were obtained based on the TRRUST database. Then, miRNAs targeting the five DE-EMGs were further explored based on miRWalk 3.0, and 55 DE-EMG-miRNA pairs were obtained. DANCR, a lncRNA related to the five DE-EMGs, was also investigated. Next, miRNAs related to DANCR were explored using DIANA-LncBasev2. Overlapping genes of these miRNAs and those involved in the 55 DE-EMG-miRNA pairs were explored, and seven miRNAs were identified, namely: hsa-miR-125b-2-3p, hsa-miR-146b-3p, hsa-miR-23b-3p, hsa-miR-380-5p, hsa-miR-4755-3p, hsa-miR-6852-5p, and hsa-miR-6889-3p. Furthermore, seven DE-EMG-miRNA pairs were obtained, with hsa-miR-23b-3p associated with osteoporosis.

Forty chemical drug molecules related to DE-EMGs were screened based on the CTD database. This included 17 small molecules, including bisphenol A, cadmium, dexamethasone, drugs, Chinese herbs, estradiol, ethanol, genistein, glyphosate, lipopolysaccharides, methotrexate, perfluoro-n-nonanoic acid, perfluorooctanoic acid, plant extracts, quercetin, resveratrol, streptozocin, and tretinoin.

**Figure 10:**
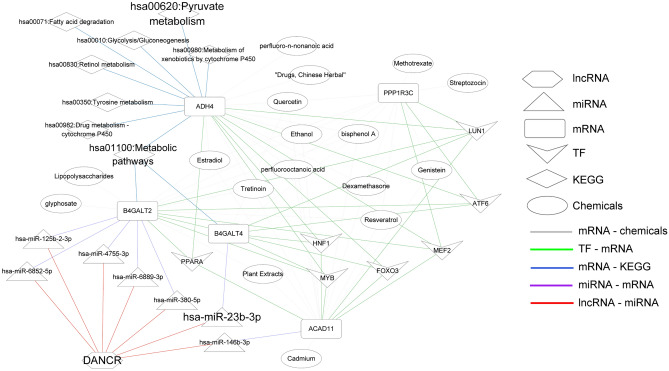
The network based on five DE-EMGs selected for constructing a diagnosis model.

Eight KEGG pathways related to the DE-EMGs were identified, including tyrosine metabolism, fatty acid degradation, and pyruvate metabolism pathways (Table 4). After comparing the 168 KEGG pathways involved in the CTD, two KEGG pathways were identified: hsa01100: metabolic pathways and hsa00620: pyruvate metabolism. The integrated network related to the five DE-EMGs and osteoporosis is shown in Fig. 10.

**Table 4:** Kyoto Encylopaedia of Genes and Genomes pathways enriched by five differentially expressed energy metabolism genes (DE-EMGs) selected for constructing diagnosis models.

Term	Count	PValue	FDR	Genes
hsa00350:Tyrosine metabolism	2	0.017438	0.067488	ADH4
hsa00071:Fatty acid degradation	2	0.020802	0.067488	ADH4
hsa00620:Pyruvate metabolism	2	0.022721	0.067488	ADH4
hsa01100:Metabolic pathways	3	0.022736	0.067488	B4GALT2, ADH4, B4GALT4
hsa00010:Glycolysis / Gluconeogenesis	2	0.032271	0.067488	ADH4
hsa00830:Retinol metabolism	2	0.032747	0.067488	ADH4
hsa00982:Drug metabolism—cytochrome P450	2	0.034647	0.067488	ADH4
hsa00980:Metabolism of xenobiotics by cytochrome P450	2	0.037494	0.067488	ADH4

## Discussion

Energy metabolism has attracted increasing attention in investigations of the pathophysiology of chronic diseases, including osteoporosis. In this study, 72 genes were selected as DE-EMGs, and a diagnostic model of five DE-EMGs were constructed: B*4GALT4, ADH4, ACAD11, B4GALT2*, and *PPP1R3C*. ROC and nomogram models confirmed good predictive ability based on the genes. Furthermore, five immune cell types showed significant differences between the high- and low-BMD groups: central memory CD8 + T cells, regulatory T cells, effector memory CD8 + T cells, activated B cells, and activated CD8 + T cells. Networks based on DE-EMGs showed that hsa-miR-23b-3p, DANCR, 17 small-molecule drugs, and two KEGG pathways, including metabolic pathways and pyruvate metabolism, might be involved in osteoporosis development.

*B4GALT4, ADH4, ACAD11, B4GALT2*, and *PPP1R3C* may be valuable biomarkers for the development of osteoporosis. ADH4, a critical member of the ADH family, is a well-known prognostic biomarker for hepatocellular carcinoma and is involved in the metabolism of ethanol and retinol. Retinol levels are associated with the occurrence of osteoporosis^35,36^. Osteoporosis might be caused by poor nutrition. The upregulation of PPP1R3C expression significantly increases glycogen synthesis and storage^37^. B4GALT family genes are associated with multiple biological processes such as cell apoptosis and proliferation^38^. Moreover, ACAD11 is required when cells undergo glucose starvation and is involved in fatty acid oxidation^39^. Thus, we propose that *B4GALT4, ADH4, ACAD11, B4GALT2*, and *PPP1R3C* are involved in the development of osteoporosis, potentially by modulating metabolic pathways.

In this study, DANCR was identified as an important lncRNA in the network based on the five selected DE-EMGs. MiR-23b-3p and DANCR act as competing endogenous RNA targeting *B4GALT4*, whose roles have been previously investigated. Yasuoka et al*.*^40^ showed that this gene was mainly involved in the regulation of notochord morphogenesis. Previous studies identified DANCR as an essential mediator of osteoblast differentiation. Further evidence demonstrates that blood mononuclear cells with upregulated DANCR expression could lead to osteoporosis, thereby increasing the secretion of IL-6 and TNF-α as well as bone resorbing activity^41^. MiR-23b-3p has been verified as a potential biomarker for osteoporosis because it participates in bone mineral density variation^42^. Moreover, the Wnt/β-catenin signaling pathway has been confirmed as a DANCR and miR-23b-3p targeted pathway in osteoporosis^43,44^. Our data predicted that metabolic pathways were enriched in the DANCR/hsa-miR-23b-3p/B4GALT4 axis. Osteoporosis is a metabolic bone disease, and its occurrence is related to various metabolic, genetic, and nutritional factors. Previous evidence has shown that a lower BMD is usually found in patients with metabolic syndrome than in those without metabolic syndrome^45^. Therefore, we hypothesized that the DANCR/hsa-miR-23b-3p/B4GALT4 axis may provide novel molecular insights into the development of osteoporosis.

The imbalance between bone formation and resorption is one of the causes of bone loss. Previous evidence has shown that bone resorption and formation can be modified by complex interactions among dendritic cells, B lymphocytes, and T lymphocytes^46^. Furthermore, several studies have confirmed the importance of T lymphocytes in the regulation of bone resorption, which might be mediated by various cytokines, such as IFN-γ, TNF-α, and IL-6^47,48^. Patients with osteoporosis have a higher CD4 + /CD8 + ratio compared to the control group^49^. Our data showed that five immune cell types, central memory CD8 T, regulatory T, effector memory CD8 T, activated B, and activated CD8 + T cells, differed significantly between the high- and low-BMD groups. These data suggest that the ratio of CD8 T cells may be used to assess osteoporosis outcomes.

Our study performed a comprehensive analysis of important EMGs in osteoporosis and developed a diagnosis signature consisting 5 EMGs, including *B4GALT4, ADH4, ACAD11, B4GALT2*, and *PPP1R3C* with a relative higher AUC*. The* differential expression of genes in the model was validated by RT-qPCR. To the best of our knowledge, this is the first study to develop a diagnosis signature based on EMGs. However, there are some limitations in this study. First, the differential expression of the five EMGs were screened from three datasets with individual heterogeneity of background and only validated in a small sample size. Second, the diagnosis signature should still be validated in large sample-size clinical studies in future. Third, the ceRNA network established in this study is warranted for in vivo or in vitro experiments.

Overall, this study constructed a model that can predict the incidence of osteoporosis and identified *B4GALT4, ADH4, ACAD11, B4GALT2*, and *PPP1R3C* as potential biomarkers for osteoporosis development. The ratio of CD8 T cells might enable the assessment of osteoporosis outcomes. Furthermore, the DANCR/hsa-miR-23b-3p/B4GALT4 axis may provide novel molecular insights into osteoporosis development. However, further clinical studies with more participants should be conducted to verify this conclusion.

## Data Availability

The datasets used and/or analysed during the current study available from Gene Expression Omnibus (GEO; https://www.ncbi.nlm.nih.gov/) with accession number of GSE56814, GSE62402, and GSE7158.

## Abbreviations

BMD: Body mineral density
DE-EMGs: Differentially expressed energy metabolism genes
KEGG: Kyoto Encyclopedia of Genes and Genomes
GEO: Gene Expression Omnibus
GO: Gene ontology
BP: Biological processes
CC: Cellular components
MF: Molecular functions
WGCNA: Weighed gene co-expression network analysis
ROC: Receiver operator characteristic
GSEA: Gene set enrichment analysis
LASSO: Least absolute shrinkage and selection operator
